# Rhesus rotavirus NSP1 mediates extra-intestinal infection and is a contributing factor for biliary obstruction

**DOI:** 10.1371/journal.ppat.1012609

**Published:** 2024-09-30

**Authors:** Enkai Li, Ningguo Feng, Qiru Zeng, Liliana Sanchez-Tacuba, Takahiro Kawagishi, Grace Branham, Gaopeng Hou, Zemin Wang, Harry B. Greenberg, Siyuan Ding

**Affiliations:** 1 Department of Molecular Microbiology, Washington University School of Medicine, St. Louis, Missouri, United States of America; 2 Department of Medicine, Division of Gastroenterology and Hepatology, Stanford University School of Medicine, Stanford, California, United States of America; 3 Department of Microbiology and Immunology, Stanford University School of Medicine, Stanford, California, United States of America; 4 VA Palo Alto Health Care System, Department of Veterans Affairs, Palo Alto, California, United States of America; 5 Department of Virology, Research Institute for Microbial Diseases, Osaka University, Osaka, Japan; Universidad Nacional Autonoma de Mexico Direccion General de Bibliotecas: Universidad Nacional Autonoma de Mexico, MEXICO

## Abstract

We previously demonstrated that in *Ifnar1*^-/-^*Ifngr1*^-/-^ or *Stat1*^-/-^ suckling mice lacking intact type I and type II interferon (IFN) signaling, rhesus rotavirus (RRV) infection causes a lethal disease with clinical manifestations similar to biliary atresia, including acholic stools, oily fur, growth retardation, and excess mortality. Elevated levels of viral RNA are detected in the bile ducts and liver of diseased pups together with severe inflammatory responses in these tissues. However, the viral determinants and the molecular mechanisms driving this process remain incompletely understood. Using an optimized rotavirus (RV) reverse genetics system, we generated a panel of recombinant RVs that encode non-structural protein 1 (NSP1) derived from different RV strains. We found that compared to the parental simian SA11 strain that is less biliary pathogenic, SA11 containing an RRV-derived NSP1 resulted in severe biliary obstructive disease comparable to that associated with RRV infection, reflected by high levels of viral RNA and inflammation in the biliary tract, liver, and pancreas. In contrast, RRV containing an SA11-originated NSP1 showed only mild biliary obstruction comparable to what was observed during SA11 infection. Infection with a monoreassortant RRV virus carrying NSP1 from the bovine RV UK strain also showed substantially reduced viral replication in extra-intestinal organs and did not develop clinical biliary diseases. Mechanistically, RRV NSP1 seemed to promote active viral replication in hepatocytes and this expanded tropism led to enhanced infiltration of CD4 and CD8 T cells, causing immunopathology and damage in the hepatobiliary system. These results highlight an unexpectedly important role of RV NSP1 in viral replication and disease progression in extra-intestinal tissues.

## Author summary

In our research, we investigated how specific proteins from the rotavirus, a virus known for causing gastrointestinal infections, influence the severity of liver diseases in a special mouse model. We found that a protein called NSP1, which varies between different strains of the virus, plays a key role in how severe the liver disease becomes.

Mice that lacked certain immune system components developed a serious liver condition similar to biliary atresia—a disease that affects the bile ducts and liver. When these mice were infected with a strain of rotavirus carrying NSP1 from another strain, they showed much more severe liver damage compared to those infected with a different strain.

Our findings highlight the significant role of NSP1 in promoting viral replication in the liver and bile ducts. This research may help us understand whether and why some rotavirus strains cause more severe liver disease than others and could guide future studies aiming to develop better treatments for such infections. By uncovering these details, we aim to improve our understanding of rotavirus and its impact on the liver, potentially leading to better strategies for managing and treating related diseases.

## Introduction

Group A rotaviruses (RVs) are the single most common cause of severe infantile diarrhea that results in over 200,000 deaths annually, predominantly in resource-limited countries [1]. RV primarily replicates in mature absorptive enterocytes of the small intestine. However, viral dissemination to the circulation and to several extra-intestinal organs has been reported [2]. Systemic disease in people due to RV infection is rare but human RV infection has been associated with encephalitis, acute pancreatitis, hepatic abscess, acute renal failure, meningoencephalitis, encephalopathy, myocarditis, mild elevation of liver transaminase levels, and pneumonitis [3–8]. In a suckling mouse model of experimental infection, RVs have been detected in several extra-intestinal organs such as liver, mesenteric lymph nodes, spleen, kidney, brain and, most frequently, the systemic circulation [9]. Riepenhoff-Talty *et al*. first reported that 2-day-old Balb/c mice orally infected with a simian RV RRV strain developed a biliary obstructive condition characterized by bilirubinuria, acholic stools, jaundice, and an extrahepatic biliary obstruction, eventually resulting in high mortality in infected pups [10]. Later, it was shown that intraperitoneal (i.p.) inoculation of RRV in newborn Balb/c mice more consistently resulted in a fatal biliary obstructive disease [11,12]. Thus, RRV infection of newborn mice via the i.p. route was generally adopted as a tractable animal model to study the etiology and pathogenesis of the human biliary atresia [13], a leading condition requiring liver transplantation in children.

Another commonly used model of lethal biliary obstructive disease involves oral inoculation of RRV in 4-5-day-old interferon (IFN) deficient mice, such as *Ifnar1*^-/-^*Ifngr1*^-/-^ or *Stat1*^-/-^ mice, paralleling what was previously observed following i.p. infection of newborn mice [14]. Deficiency in IFN signaling is necessary for the induction of biliary obstruction following oral RRV administration in this model. This is because heterologous non-murine RV strains, *e*.*g*., RRV, undergo only limited viral replication in the 4-5-day-old suckling mouse intestine, unlike homologous wild-type murine RVs, which replicate at much higher levels (> 10,000-fold) in the small intestine. This host range restriction is primarily determined by the different ability of heterologous versus homologous RVs to counteract the host antiviral IFN responses [15].

Intriguingly, another simian origin RV SA11 strain as well as the homologous wild-type murine RV EDIM-EW strain were not able to induce biliary disease [14], despite high levels of intra-intestinal replication in IFN deficient mice, providing an opportunity to dissect the specific RV genes responsible for this virulent phenotype. RV gene products NSP3 has previously been implicated in RRV associated systemic spread and bile duct infection in neonatal CD-1 mice [16]. In addition, a series of elegant work from the Tiao lab has demonstrated a critical role of VP4 protein as a molecular driver of biliary atresia in newborn Balb/c mice [17–19]. In previous studies from our lab, we observed elevated simian RRV strain replication in the liver, biliary tract, and pancreas in IFN-deficient 129sv mice [14]. By directly injecting RV into the mouse gallbladder and using *in vitro* infection in murine cholangiocyte cultures, we found that, when comparing RRV and the non-biliary pathogenic bovine RV strain UK, both the RRV VP4 and NSP1 proteins were necessary for producing the high replication phenotype of RRV in the biliary tract and cholangiocytes [20].

Taking advantaging of an optimized plasmid-based RV reverse genetic system [21], we have begun to further investigate the role of VP4 and NSP1 in RV host range [22,23]. We utilized reverse genetics to generate specific monoreassortant viruses by design and more precisely interrogate the role of specific viral products in this model of RRV-induced biliary obstructive disease in IFN deficient suckling mice. We initially drew comparisons between RRV and the low biliary pathogenic simian RV SA11 strain to generate NSP1 monoreassortants because these two heterologous viruses, both of simian origin, have similar intestinal replication and enteric disease phenotypes in suckling mice [14]. To summarize, we found that at least in our model system, RRV NSP1 but not VP4 was necessary and sufficient to induce enhanced viral replication and severe inflammation in the liver, intrahepatic biliary ducts, and pancreas in *Stat1*^-/-^ suckling mice. This activity is independent of the ability of RRV NSP1 to target host IRF3 protein for degradation. Taken together, our new results suggest that NSP1, in addition to its well-established function as an IFN antagonist [24,25] and intra-intestinal replication [22], also plays a unique role in modulating viral replication and inflammatory responses in several extra-intestinal tissues.

## Materials and methods

### Ethics statement

All animal studies were approved by Veteran Affairs Palo Alto Health Care System Research and Development Information System committee and Washington University in St. Louis Institutional Animal Care and Use Committee.

### Rotavirus infection of mice

*Stat1*^-/-^ mice (129S6/SvEv genetic background) were originally purchased from Taconic (Germantown, NY) and maintained in Veterinarian Medical Unit of Palo Alto VA Medical Center. These mice were used to generate suckling pups for these experiments. Four-to-five-day-old suckling mice were orally inoculated with 4x10^7^ plaque forming units (PFUs) of the indicated RV strains or control media. Inoculated mice were observed daily for 10- and 14-days post inoculation at Stanford University and Washington University in St. Louis, respectively, and the occurrence of diarrhea, acholic stools, and oily fur were recorded and body weights were measured daily. Stool samples were also collected for the detection of RV shedding as previously described [26]. At days 6, 8 and 10 post infection, selected mice were sacrificed and intestine, pancreas and liver (with attached extrahepatic bile ducts) were collected for the detection of virus by qRT-PCR. Pancreas and liver tissues were also collected for histology, immunohistochemistry, and immunofluorescent staining.

### Measurement of virus levels in stool and tissue samples by qRT-PCR

Procedures for viral detection and quantification by measuring RV RNA copy number by qRT-PCR were described previously [14]. Briefly, RNA from stool and tissue samples was extracted using Trizol and chloroform. Viral RNA levels were measured using qRT-PCR with primer pairs based on RV NSP5 sequences and Cy5 conjugated probes manufactured by Integrated DNA Technologies, Inc. San Diego, CA. A serially diluted NSP5 plasmid was used to construct a standard curve for calculating viral RNA copy numbers [27]. Real-time PCR was performed with an Agilent Stratagene Mx3005P Real Time PCR instrument (Agilent Technologies, Inc., Santa CA). Levels of viral RNA in stool or tissue samples were expressed as RNA copy numbers per mg of sample.

### Histology, immunofluorescent and immunohistochemistry staining and serum clinical chemistry measurements

For histology, pancreas and liver samples taken at 10 days post infection were fixed in 10% formalin. Tissues were paraffin embedded, sectioned and H&E stained by IDEXX BioAnalytics, Columbia MO. For immunofluorescence staining, fresh tissues were frozen using Tissue-Tek Cryo-OCT Optimum Cutting Temperature (OCT) Compound and cut into 7–10 μm sections with a microtome. Slides were fixed using a 1:1 of acetone and methanol mixture for 20 min. After washing, the slides were stained with Texas-red conjugated rabbit polyclonal anti-RV DLP antibody [28], Alexa Fluor 488 conjugated rabbit monoclonal antibody anti-cytokeratin 7 (EPR17078, Abcam, Waltham, MA), and DAPI. Another set of samples were stained with CK19 (1:400, ab52625, Abcam), α-SMA (1:500, ab5694, Abcam), VisUCyte HRP polymer (1:4, VC003-025, R&D), and DAB (SK-4100, Vector). CK7 and anti-RV antigen slides were observed and images collected with a Keyence BZ-X710 All-in-one fluorescence microscope. CK19 and SMA slides were visualized with an ECHO Revolve microscope (Discover Echo, San Diego, CA, USA).

### Clinical chemistry tests for serum samples

Mouse serum samples were collected at day 10 post infection for clinical chemistry testing. Conjugated bilirubin, pancreatic enzyme lipase and amylase as well liver enzyme alanine aminotransferase (ALT) were measured by IDEXX BioAnalytics (West Sacramento, CA). Levels of conjugated bilirubin were expressed as mg/dL. Levels of lipase, amylase, and ALT were expressed as U/L.

### RV reverse genetics system

Recombinant SA11 and RRV viruses was generated as follows: 2 × 10^5^ BHK-T7 cells were seeded into 1 well of 12-well plate with 1 ml of complete DMEM (10% heat-inactivated FBS, 100 IU/ml penicillin, 100 μg/ml streptomycin, 0.292 mg/ml) G418-free medium. Twenty-four hours later, the medium was replaced by 800 μl of fresh complete DMEM medium, and then the sub-confluent BHK-T7 monolayer was transfected with the corresponding transfection mix, which contained 125 μl of prewarmed Opti-MEM, 400 ng each of pT7-VP1-7, pT7-NSP1,3,4 and 1200ng pT7-NSP2,5, and 800 ng of the plasmid C3P3-G1 together with 14 μl of TransIT-LTI (Mirus Bio LLC). All the plasmids and transfection reagents were mixed in a pipet by gently moving them up and down and then incubated at room temperature for 15 min. Transfection mixture was added drop by drop to the medium of BHK-T7 monolayers, and then the cells were returned to 37°C. 18 h later, two washes with FBS-free medium, after that 800 μl of serum-free DMEM was added to the transfected-BHK-T7 cells. Twenty-four hours later, 5 × 10^4^ MA104 N*V cells in 200 μl of serum-free DMEM was added to the well, along with 0.5 μg/ml of porcine pancreatic type IX-S trypsin (Sigma-Aldrich). MA104 N*V cells and BHK-T7 cells were co-cultured for 72 h, after which they were frozen and thawed three times as previously described [29]. To generate RRV with NSP1 from bovine RV UK strain, gene segment 5 of UK strain was cloned from the virus stock amplified in MA104 cells by PCR and replaced with ORF of NSP4 in pT7-SA11-NSP4 plasmid. To generate SA11 with RRV NSP1 deletion mutants (NSP1-deletion virus and ΔC16 virus), nucleotide sequences for amino acids 17 to 494 or 478 to 494 of RRV NSP1 were deleted by inverse PCR.

### Western blot

Cells were washed with PBS and lysed by RIPA buffer (Thermo Scientific, 89901) supplemented with 100X protease inhibitor cocktail and phosphatase inhibitor (Thermo Scientific, 78420), followed by a 10-minute incubation on ice. Cell lysates were then subjected to centrifugation at 13,500 RPM for 10 minutes at 4°C to remove cell debris and chromatids. The protein samples were then boiled in 2X Laemmli Sample Buffer (Bio-Rad, #1610737EDU) containing 5% β-mercaptoethanol at 95°C for 5 minutes. Prepared samples were run in 4–12% gels and transferred onto nitrocellulose membranes. Membranes were blocked in 5% BSA in TBS + 0.1% Tween-20 (TBST) at room temperature before incubation at 4°C overnight with primary antibodies to: IRF3 (1:3000, Ab68481, Abcam), VP6 (1:1000, sc-101363, Santa Cruz), and GAPDH (1:1000, 2118S, Cell Signaling Technology). Membranes were then washed three times with TBST and incubated in secondary antibodies accordingly: anti-mouse HRP-linked IgG (Cell Signaling Technology, 7076S) or anti-rabbit HRP-linked IgG (Cell Signaling Technology, 7074S) diluted in 5% BSA in TBST at room temperature for 1 hour. After the secondary antibody incubation, the membranes were washed three times with TBST and visualized by using ECL Substrates for Western Blotting (Bio-Rad). Images were acquired with Chemi-Doc imaging system (Bio-Rad).

### Immune cell profiling

Immune cell populations in the liver were harvested and stained with live/dead (zombie aqua), CD45-V450, CD4-PE/Cy7, CD8a-PE, CD19-APC, and NK1.1-BV605 (BioLegend) using BD Aria II and analyzed by FlowJo v10.10.

### Statistical analysis

All statistical analyses were performed using IBM SPSS (Chicago, IL). Animal body weights from days 6–10 post infection in different mouse pup groups were analyzed using ANOVA plus LSD post hoc test for pairwise comparisons. For analysis of fecal rotaviral shedding RNA copy numbers were log transformed. The area under the day 2–10 post infection shedding curves of different groups were analyzed using ANOVA plus LSD post hoc test for pairwise comparisons. Levels of tissue virus (measured as RNA copy numbers per mg tissue) were also log transformed. Viral levels at days 6, 8 and 10 post infection in pancreas and liver of the different experimental groups were analyzed using ANOVA plus LSD post hoc test for pairwise comparisons. For serum clinical chemistry tests, levels of bilirubin, lipase, amylase and ALT were combined for viruses with RRV NSP1 (rRRV and rSA11 with RRV NSP1) and viruses with SA11 NSP1 (rSA11 and rRRV with SA11 NSP1) in order to increase sample size. Analysis of the differences in different groups were performed using the Student’s t test.

## Results

### RRV NSP1 expression correlates with systemic clinical symptoms following RV infection of *Stat1*^-/-^ suckling mice

**Fig 1:**
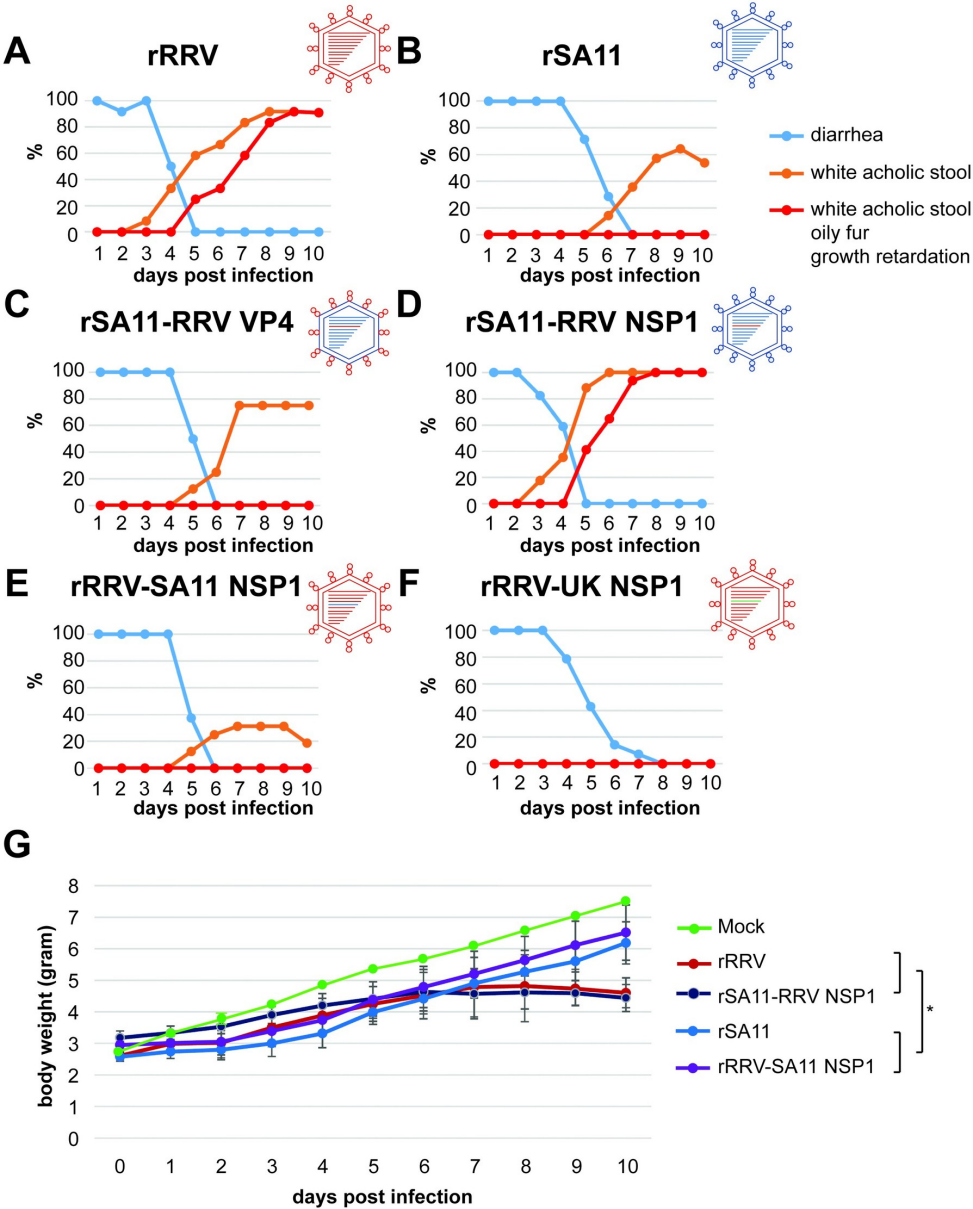
Percentage of diarrhea, acholic stool, and biliary obstructive disease (acholic stool, oily fur, and growth retardation) in *Stat1*^-/-^ mice suckling mice orally infected with rRRV, rSA11, and mono-reassortant RVs with different NSP1 or VP4 encoding genes. (A) rRRV; (B) rSA11; (C) rSA11 with RRV VP4; (D) rSA11 with RRV NSP1; (E) rRRV with SA11 NSP1; (F) rRRV with UK NSP1. N = 8–12 in each group. (G) Daily weight changes in *Stat1*^-/-^ suckling mice orally infected with mock, rRRV, rSA11 and reciprocal NSP1 reassortants on indicated days following RV infection. * (P<0.05, rRRV or rSA11-RRV NSP1 *vs*. rSA11 or rRRV-SA11 NSP1). Without indication, P>0.05, not significant.

Four-to-five-day-old *Stat1*^-/-^ suckling mice were orally inoculated with a panel of reverse genetics derived recombinant (r) RVs and clinical symptoms and body weights were recorded daily (**Fig 1**). For the parental rRRV and rSA11 viruses, all infected mice experienced diarrhea, which resolved between 5–7 days post infection (**Fig 1A and 1B).** Starting on day 3 post inoculation, rRRV infected suckling mice developed white-colored acholic stools. 92% of mice in the rRRV group had acholic stools on 8 days post infection. From day 5 post infection, rRRV infected suckling mice also developed more severe systemic symptoms manifest as oily fur and growth retardation in addition to acholic stools, consistent with the development of more severe biliary obstructive diseases (**Fig 1A and 1G**). These mice eventually succumbed to infection, which had been previously noted following RRV i.p. inoculation in newborn mice [11,14]. In contrast, rSA11 infected suckling mice developed more mild biliary obstructive symptoms, including only acholic stool that started at day 6 post infection and reached 60% penetrance by day 9 post infection (**Fig 1B**). In addition, no severe symptoms, such as oily fur and growth retardation, were observed in rSA11 infected mice (**Fig 1B and 1G**). The distinctive disease phenotypes in this study were consistent with previously described RRV and SA11 infection symptoms seen in *Ifnar1*^-/-^*Ifngr1*^-/-^ infected suckling mice [14].

To identify the genetic basis of the distinct systemic disease phenotype seen with RRV infection, we leveraged the RV reverse genetics system and generated two monoreassortants using an SA11 genetic backbone expressing either RRV VP4 or NSP1. *Stat1*^-/-^ suckling mice infected with SA11 encoding RRV VP4 behaved similar to those infected with rSA11 (**Fig 1C**), suggesting that RRV VP4 was not correlated with the biliary disease phenotype in these experiments. In contrast, we found diarrheal disease, acholic stools, oily fur, and significantly reduced weight gain in mice infected with the other monoreassortant, rSA11 encoding an RRV NSP1. These mice behaved similarly to the parental rRRV infected mice (**Fig 1D and 1G**). Importantly, *Stat1*^-/-^ suckling mice orally inoculated with the reciprocal reassortant, an rRRV encoding an SA11 NSP1, developed similar diarrhea and acholic stools to the rSA11 infected pups with no oily fur, weight loss or failure to thrive (**Fig 1E and 1G**). Further, we generated an additional monoreassortant on the RRV backbone expressing NSP1 derived from the bovine origin UK strain, which does not cause any biliary diseases. *Stat1*^-/-^ suckling mice orally inoculated with this recombinant virus did not have any systemic disease manifestations besides diarrhea (**Fig 1F**). Taken together, these data suggest that the penetrance and severity of systemic disease caused by RV infection in mice co-segregates with the genetic origin of NSP1.

### The C-terminal region of RRV NSP1 is important for the biliary disease development

**Fig 2:**
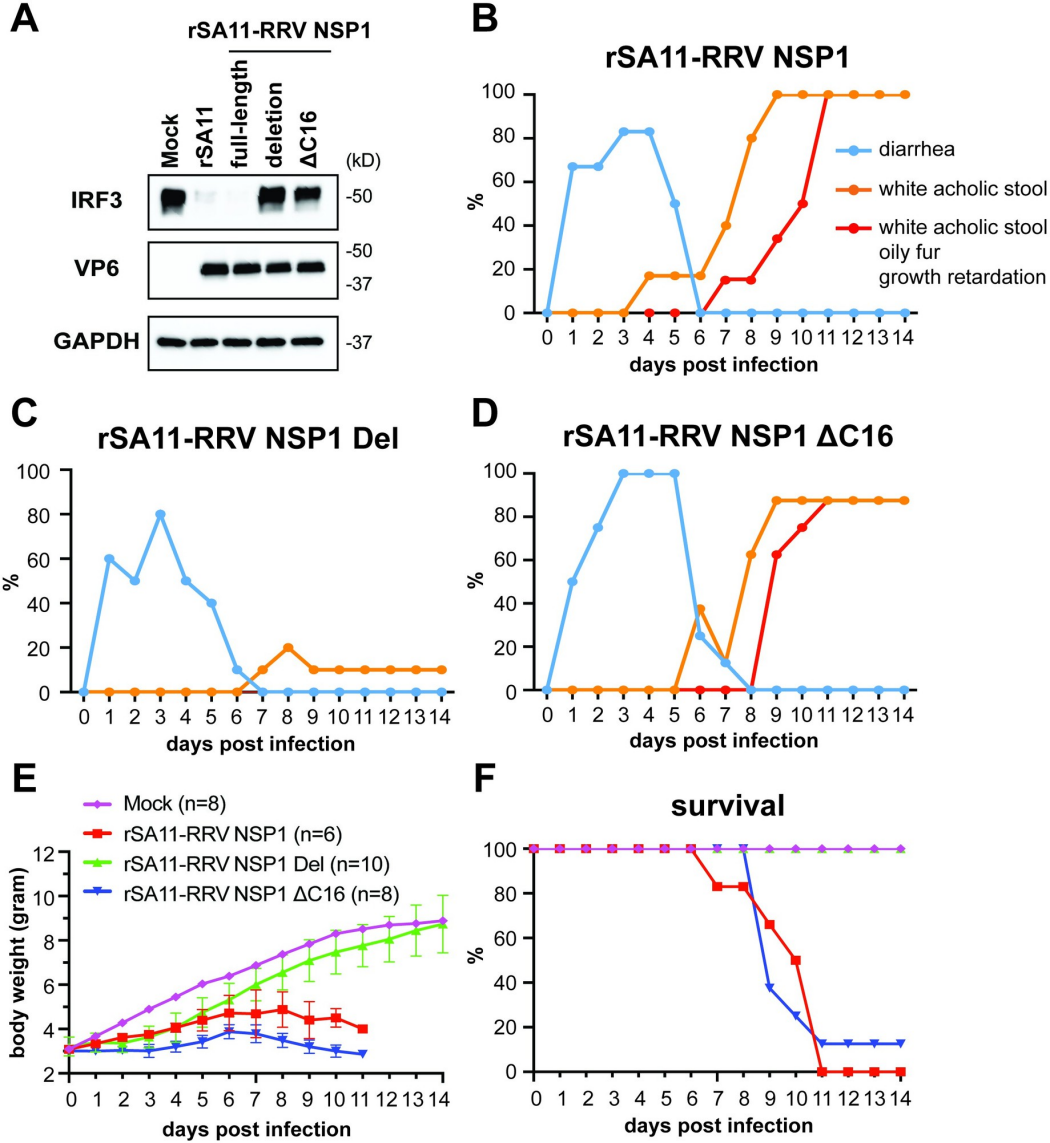
Percentage of diarrhea, acholic stool, and biliary obstructive disease (acholic stool, oily fur, and growth retardation) in *Stat1*^-/-^ mice suckling mice orally infected with recombinant RVs encoding different regions of RRV NSP1. (A) Western blot images of IRF3 degradation in MA104 cells (multiplicity of infection of 3, 8 hours post infection). (B) rSA11 with RRV NSP1; (C) rSA11 with RRV NSP1 deletion; (D) rSA11 with RRV NSP1 16 amino acids truncated at the C-terminal end; (E) daily weight changes; (F) Kaplan–Meier survival curves. Del: deletion.

RRV NSP1 abrogates host IFN induction by targeting IRF3 for proteasomal degradation [30]. To determine if the IFN antagonistic activity of NSP1 is required for biliary disease, we further used the reverse genetic system and generated two more recombinant SA11 viruses that encode different regions of the RRV NSP1 protein. One expressed only the first N-terminal 16 amino acids (termed NSP1-deletion virus) and the other lacked the last C-terminal 16 amino acids critical for IRF3 degradation, with amino acid L479 replaced with a premature stop codon [31] (termed ΔC16 virus). As expected, compared to the parental SA11 virus and SA11 encoding full-length RRV NSP1, both NSP1-deletion and ΔC16 viruses failed to induce IRF3 degradation *in vitro* (**Fig 2A**). In suckling mice, compared to the parental SA11 and SA11 expressing full-length NSP1, NSP1-deletion virus infection did not cause biliary atresia, confirming that RRV NSP1 is critical for the disease progression (**Fig 2B and 2C**). Notably, ΔC16 virus infection led to similar penetrance of acholic stool, oily fur, weight loss, and mortality as the SA11 virus expressing RRV NSP1 (**Fig 2D–2F**), suggesting that an uncharacterized domain of NSP1 other than the IRF3-binding motif is involved in the extra-intestinal pathogenesis.

To further interrogate the non-IRF3 binding domains of RRV NSP1 that contribute to the biliary obstruction, we constructed and rescued a series of rSA11 that encode truncated RRV NSP1 mutants R86*, C179* and N328* to determine the functional role of RING-finger domain, cytoskeleton binding domain, and an undefined domain (25). We then examined their ability to cause white acholic stool, oily fur, and growth retardation in neonatal *Stat1*^-/-^ mice. Interestingly, none of these NSP1 truncation mutants caused biliary atresia or lethality (**S1 Fig**). These data suggest that the region between amino acids N329 and K478 within RRV NSP1 is required for viral pathogenesis in this context.

### RRV NSP1 enhances viral presence in extra-intestinal tissues

**Fig 3:**
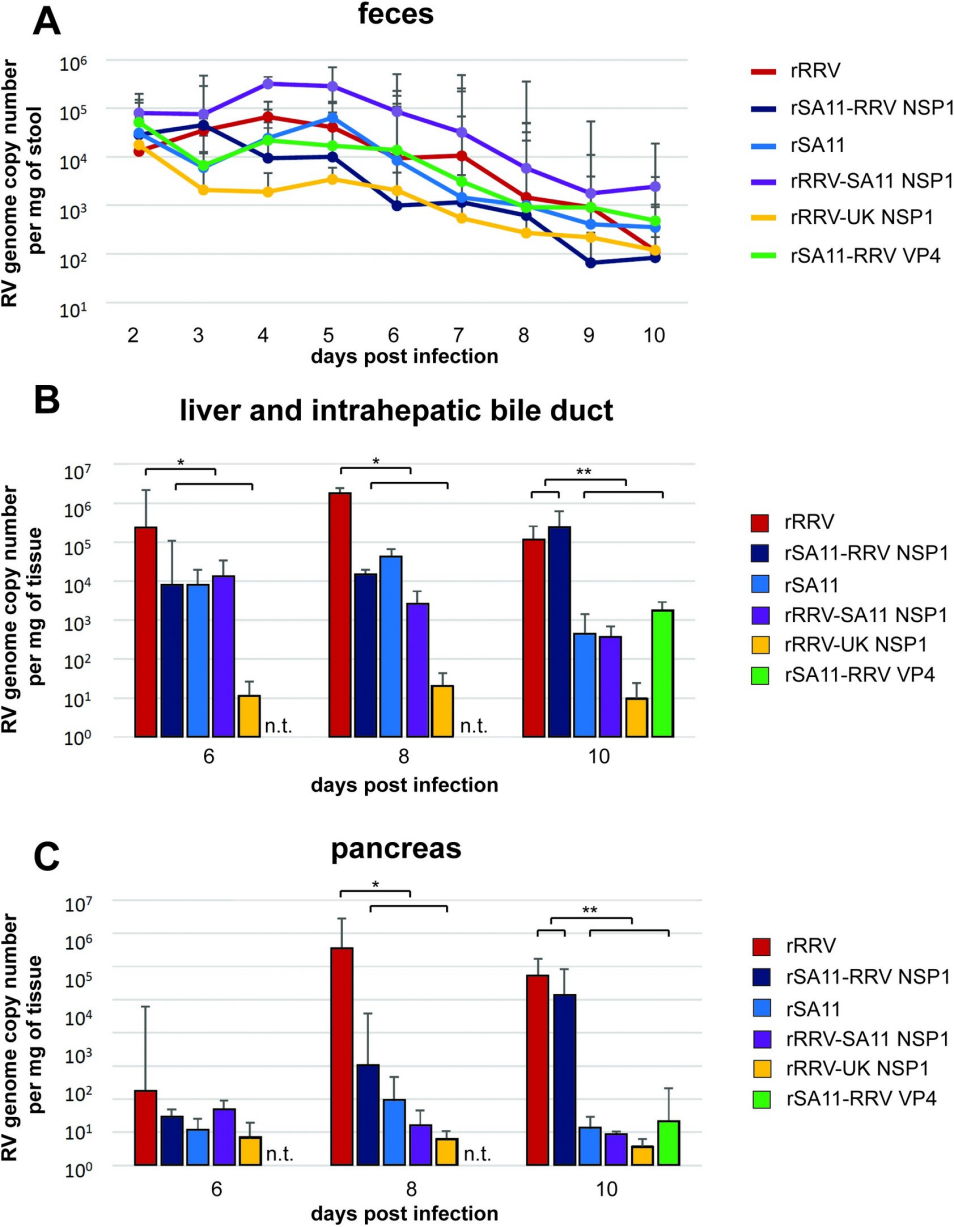
Fecal virus shedding and levels of viral replication in liver and pancreas in *Stat1*^-/-^ suckling mice orally infected with rRRV, rSA11, and mono-reassortant RVs with different NSP1 or VP4 encoding genes. (A) Fecal virus shedding; (B) levels of viral replication in livers; (C) levels of viral replication in pancreases. Brackets show statistically significant difference between the viral strains and groups. * (P<0.05), ** (P<0.01). n.t., not detected.

To examine the underlying mechanism of RRV NSP1 associated biliary disease, we next determined the viral shedding in feces and viral loads in systemic organs of the selected monoreassortants. The intra-intestinal replication of parental RVs and monoreassortants did not vary significantly, as revealed by fecal RV shedding, measured by qRT-PCR (**Fig 3A**).

Next, we quantified the levels of viral replication in the liver and pancreas on days 6, 8 and 10 post infection in mice infected with either parental rRRV or rSA11 or selected NSP1 or VP4 monoreassortant viruses. Importantly, the rRRV viral load in the liver at day 8 post infection was significantly higher than all other groups (**Fig 3B**). The amount of rRRV in the pancreas was also higher than that in all other groups on day 8 post infection (**Fig 3C**). Remarkably, by day 10 post infection, the replication of rSA11 encoding RRV NSP1 in both liver and pancreas caught up to levels similar to those of rRRV and was significantly higher than the rest of the groups (**Fig 3B and 3C**). In contrast, the replication of rSA11, rSA11 with RRV VP4, rRRV with SA11 NSP1, and rRRV with UK NSP1 in both tissues was significantly lower than the viruses encoding RRV NSP1 (**Fig 3B and 3C**).

### RRV NSP1 expands viral tropism in the liver

**Fig 4:**
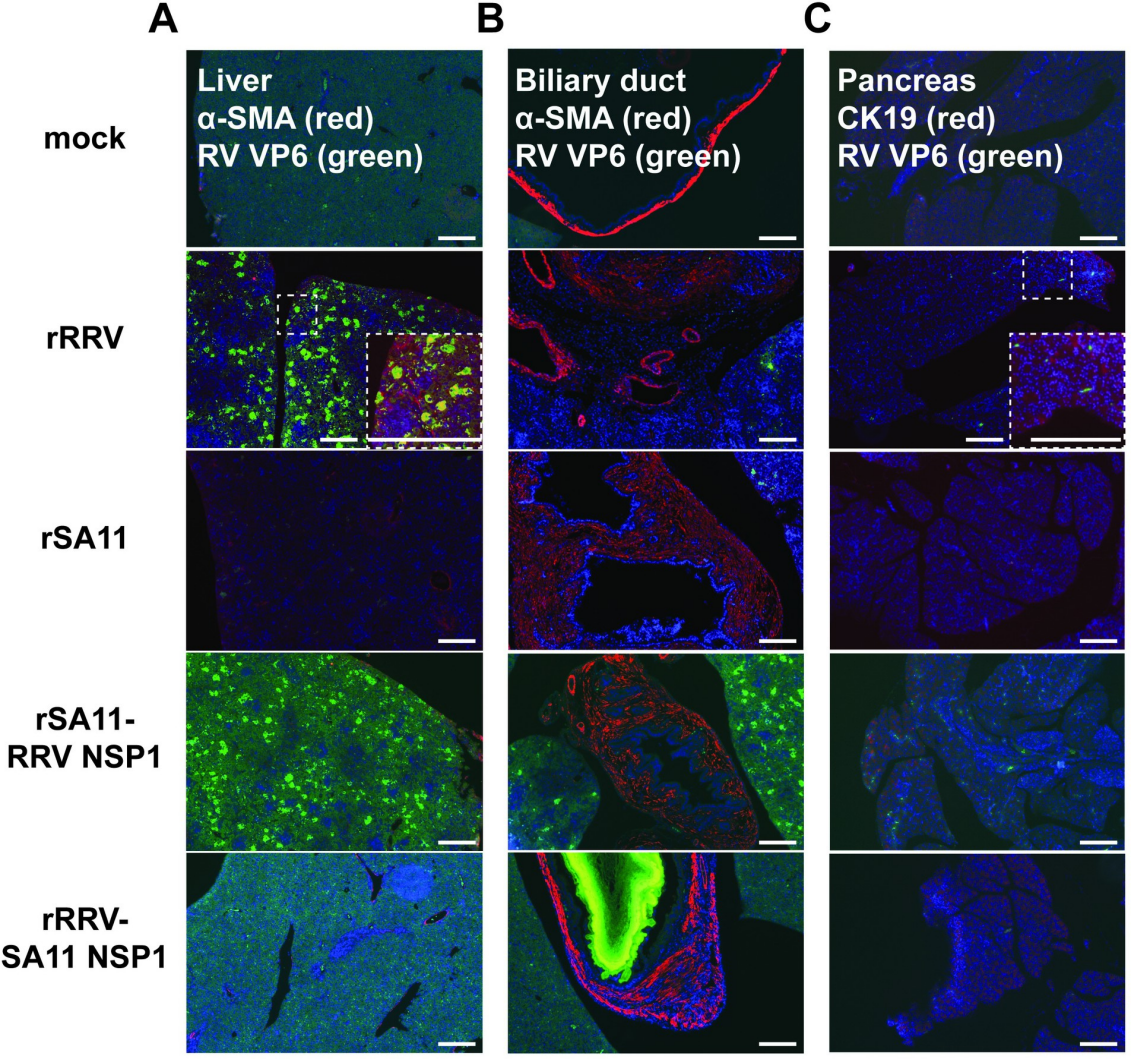
Immunofluorescent staining of liver, bile duct, and pancreas of *Stat1*^-/-^ suckling mice orally infected with mock, rRRV, rSA11 and reciprocal NSP1 mono-reassortants at day 10 post infection. Tissues were stained with Alexa Fluor 488 labeled mouse monoclonal antibody against RV VP6 (green), Alexa Fluor 594 labeled rabbit monoclonal antibodies against α-SMA or CK19, and DAPI (blue). (A) liver; (B) biliary duct; (C) pancreas. Scale bar: 1 mm.

We next asked whether the presence of RRV NSP1 enables the ability of RV to infect distinct cell types in these hepatic and pancreatic tissues. Overall, we observed that the majority of the infection from RRV NSP1-encoding viruses occurred in the liver (**Fig 4A**). Viral antigen was minimally found in alpha-smooth muscle actin (α-SMA) positive hepatic stellate cells, consistent with previous reports of RRV infection in liver portal triad area and hepatocytes [14,32]. In contrast, we did not find RV antigen staining in the biliary duct (**Fig 4B**). Similarly, relatively little infection was found in the cytokeratin 19 (CK19) positive pancreatic epithelium (**Fig 4C**).

Next, we stained for RV VP6 antigen in rRRV infected liver portal triads. Viral antigen VP6 only partially co-localized with CK7, a marker specifically found in pancreatic ductal and hepatic bile duct epithelial cells [33] (**S2A Fig**). Most RV infected cells were found in poral triad areas surrounding intrahepatic bile ducts and extended from the triads into hepatocyte areas (**S2A Fig**). Significantly less virally infected cells were observed in portal triad and nearby hepatocyte area in rSA11 infected livers (**S2B Fig**). These data are consistent with the tissue viral loads (**Fig 3B**) and gross antigen staining patterns (**Fig 4A**) and suggest that the severe biliary disease seen in RV infection (**Fig 1**) is likely due to direct viral cytopathic effect and the inflammation in extra-hepatic bile ducts and the liver portal triad, or both.

More RV infected cells were detected in pancreatic tissues of rRRV infected mice relative to rSA11 infected mice (**S2C and S2D Fig**). As in the liver specimens, viral antigens were only partially co-localized with CK7, a pancreatic ductal cell marker. The majority of RV positive cells were found in areas surrounding the ducts with a small number of infected cells also observed in the pancreatic parenchyma (**S2C Fig**). Of note, RV replication was only detected in the pancreas of rRRV but not in rSA11 infected pups (**Fig 3C**). These findings are consistent with our previous publication of RRV replication in the pancreas of IFN deficient suckling mice [14]. Therefore, the distinctive pathologic features were found in the liver and pancreas of rRRV but not in rSA11 infected mice.

### RRV NSP1 is associated with increased inflammation in the biliary ducts, liver, and pancreas

Because the difference in virological phenotypes between SA11 and SA11 expressing RRV NSP1s did not appear until 10 days post infection (**Fig 3B and 3C**), a few days later than the pathological manifestation (**Fig 1**), we next examined the effects of RRV NSP1 on extraintestinal pathological changes in the liver, biliary system, and pancreas following enteric infection with parental rRRV, rSA11, or the NSP1 monoreassortant viruses on 10 days post infection.

**Fig 5:**
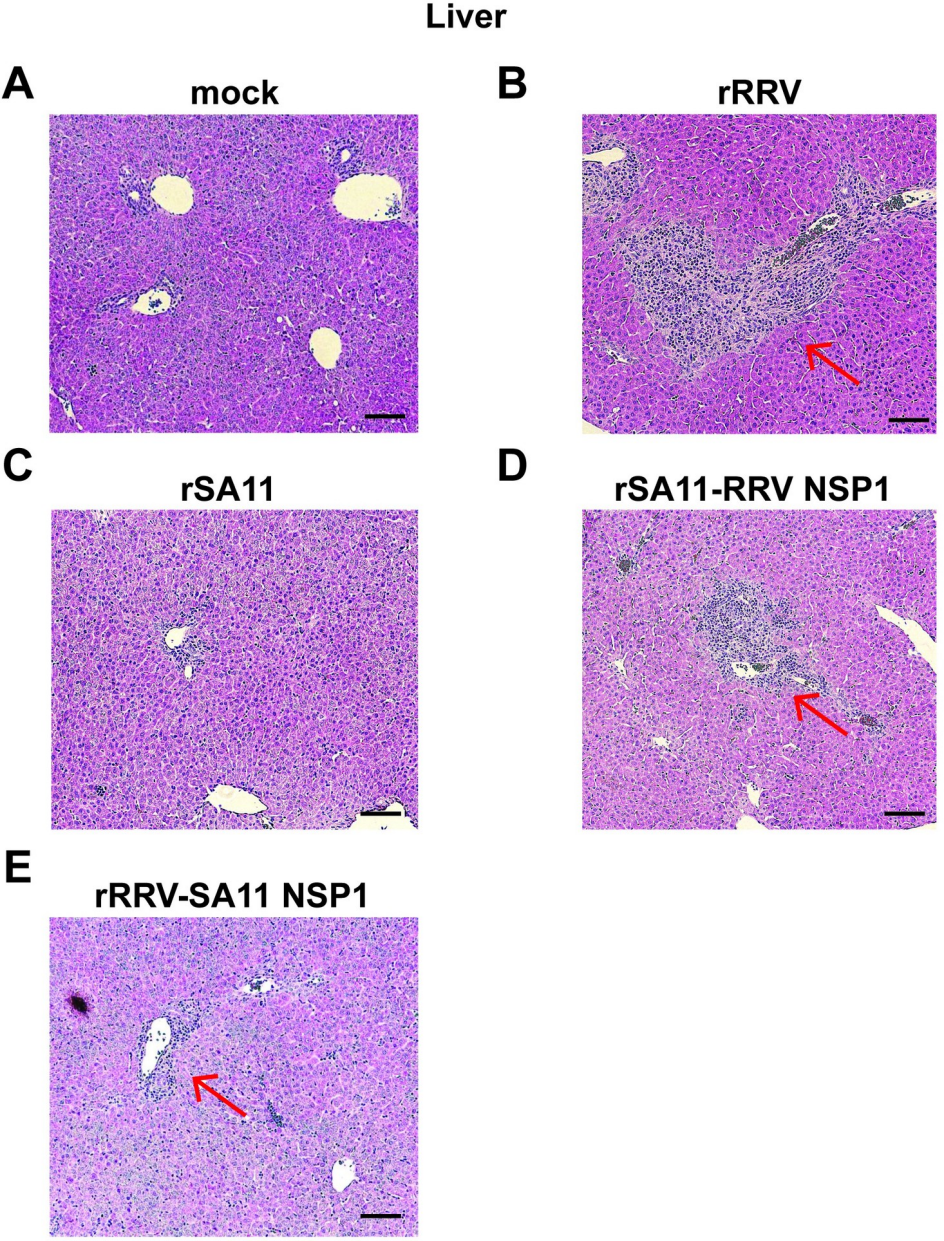
H&E staining of liver in *Stat1*^-/-^ suckling mice orally infected with rRRV, rSA11 and reciprocal NSP1 mono-reassortants at day 10 post infection. (A) uninfected mouse; (B) rRRV; (C) rSA11; (D) rSA11 with RRV NSP1; (E) rRRV with SA11 NSP1. Red arrows indicate areas of inflammation in region of portal triads. Scale bar: 200 μm.

As compared to uninfected controls (**Fig 5A**), in the livers of rRRV or rSA11 with RRV NSP1 infected pups, large areas of inflammation surrounding portal triad were observed (**Fig 5B and 5D**). Immune cells were possibly recruited from the periphery, as they were clustered and blocked the portal vein and bile ducts (**Fig 5B and 5D**). In comparison, the inflamed areas were significantly smaller in livers of rSA11 or rRRV with SA11 NSP1 infected mice (**Fig 5C and 5E**). Using flow cytometry, we did observe significant increases in the numbers of CD4 and CD8 positive T cells in the liver of mice infected by SA11 expressing RRV NSP1 (**S3A and S3B Fig**), suggesting that the bile duct obstruction is at least partly correlated with the infiltration of CD4 and CD8 positive lymphocytes. By contrast, NK cell and B cell populations were not elevated comparing SA11 and RRV-derived NSP1s (**S3C and S3D Fig**).

**Fig 6:**
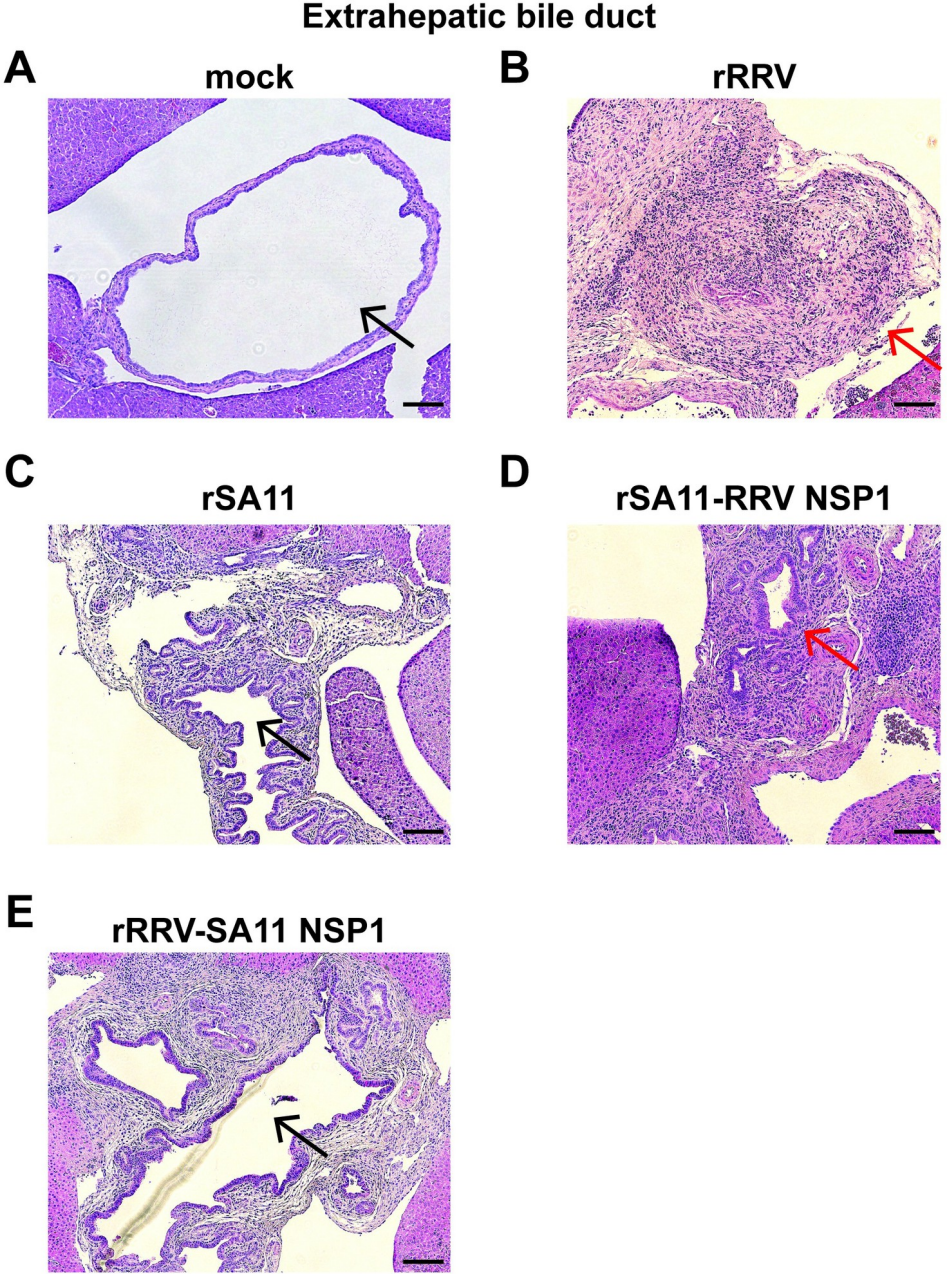
H&E staining of extra-hepatic biliary ducts in *Stat1*^-/-^ suckling mice orally infected with rRRV, rSA11 and reciprocal NSP1 mono-reassortants at day 10 post infection. (A) uninfected mouse; (B) rRRV; (C) rSA11; (D) rSA11 with RRV NSP1; (E) rRRV with SA11 NSP1. Black arrows indicate open bile ducts and red arrows indicate areas of severe biliary obstruction. Scale bar: 200 μm.

At least two studies have shown that the kinetics of RRV infection are different between the hepatic and biliary tissues. The virus seems to be cleared in the liver before the development of biliary atresia but still triggers an inflammatory response that causes the fibrosing destruction and blockage of extrahepatic bile duct [44,45]. In our study, mice infected with rRRV also had severe inflammation in the extra hepatic biliary ducts as compared to uninfected control (**Fig 6A and 6B**). Edema and immune cell infiltration surrounded the bile duct epithelial cells was observed, resulting in the narrowing and blockage of bile ducts (**Fig 6B**), which had previously been described in newborn and IFN deficient suckling mice [10,11,14]. Inflammation, although observed, was less severe in extrahepatic biliary tracts following rSA11 infection (**Fig 6C**). Importantly, infection of rSA11 with RRV NSP1 and rRRV with SA11 NSP1 led to similar amounts of severe extrahepatic bile duct inflammation as mice infected with RRV and SA11, respectively (**Fig 6D and 6E**), suggesting that the parental origin of NSP1 correlates with these biliary and pancreatic disease manifestation. The present but diminished inflammation seen with SA11 NSP1 containing viruses is likely responsible for the acholic stools observed in suckling mice infected with these constructs (**Fig 1B, 1C and 1E**). Considering the low levels of RV infection in the biliary system (**Fig 4B**), the immune cells recruited to the extra hepatic areas could be derived from the proximal inflamed liver tissues.

**Fig 7:**
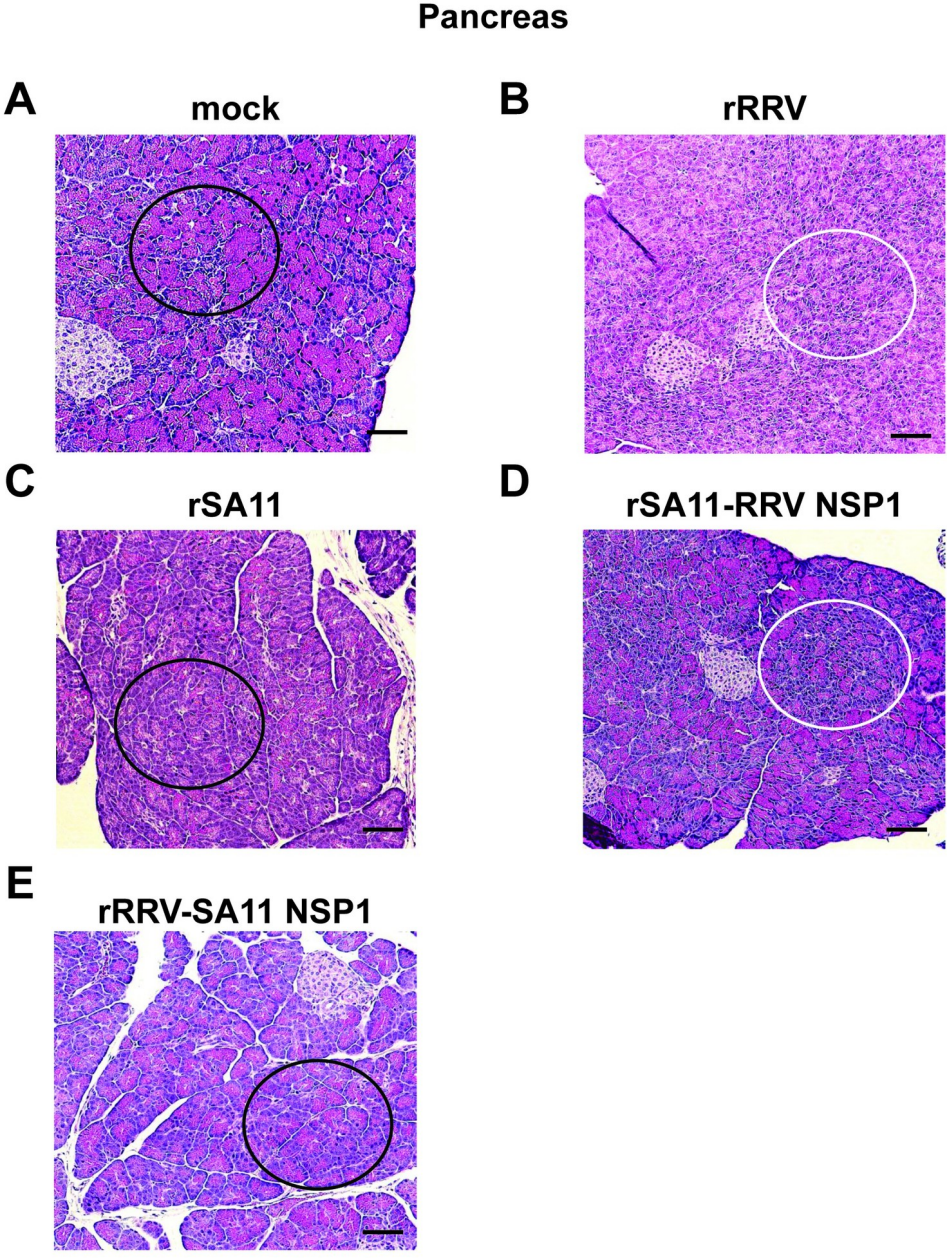
H&E staining of pancreas in *Stat1*^-/-^ suckling mice orally infected with rRRV, rSA11 or reciprocal NSP1 mono-reassortants at day 10 post infection. (A) uninfected mouse; (B) rRRV; (C) rSA11; (D) rSA11 with RRV NSP1; (E) rRRV with SA11 NSP1. Black circles indicate healthy pancreatic parenchyma and white circles indicate focal areas of inflammation. Scale bar: 200 μm.

Consistent with our findings in the bile duct and liver, as opposed to the healthy pancreatic tissue in the mock infected animal (**Fig 7A**), areas of inflammatory responses, marked by immune cell infiltration and acinocyte degeneration and necrosis, were also observed in pancreas of rRRV and rSA11 with RRV NSP1 infected mice (**Fig 7B and 7D**). The overall structural organization of the pancreas in rSA11 and rRRV with SA11 NSP1 infected mice appeared normal (**Fig 7C and 7E**), in line with the absence of detectable virus in these tissues (**Fig 3C**).

### Serum chemical assays indicate potential liver and/or pancreas injury following infections of RRV NSP1 expressing viruses

**Fig 8:**
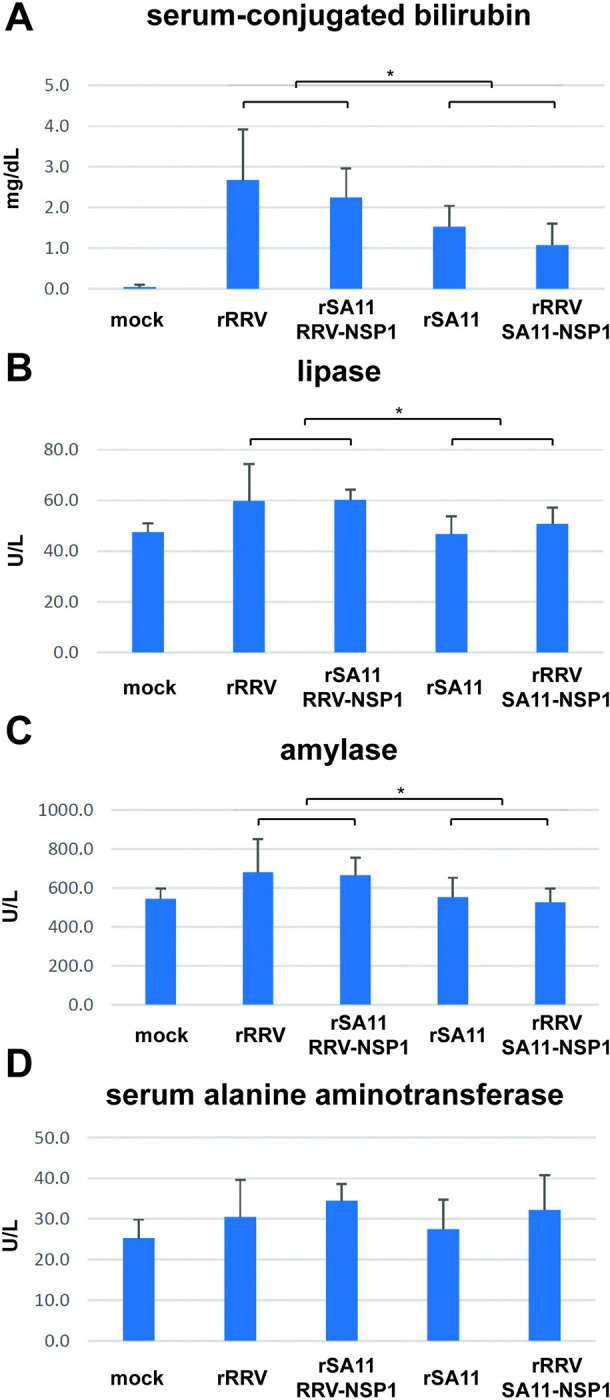
Blood chemistry analysis in serum of *Stat1*^-/-^ suckling mice orally infected with rRRV, rSA11 and reciprocal NSP1 mono-reassortants. (A) serum conjugated bilirubin; (B) serum lipase; (C) serum amylase; (D) serum alanine aminotransferase (ALT). Brackets show statistically significant differences between combined groups with RRV NSP1 vs SA11 NSP1. * (P<0.05).

Finally, we performed a series of clinical chemistry assays on serum samples from pups at 10 days post RV infection to provide biochemical evidence related to our clinical and pathological findings. When we combined serum samples of groups infected with viruses containing RRV NSP1 (rRRV, rSA11 with RRV NSP1) and groups infected with viruses expressing SA11 NSP1 (rSA11, rRRV with SA11 NSP1), the levels of serum conjugated bilirubin, lipase, and amylase in mice infected with RRV NSP1 viruses were significantly higher than in mice infected with SA11 NSP1 expressing viruses (**Fig 8A–8C**). Unexpectedly, the level of serum alanine aminotransferase (ALT), a marker of hepatocyte damage, on the other hand, was similar between mock and all the infected groups (**Fig 8D**). The elevations of serum bilirubin, lipase, and amylase in mice infected with the RRV NSP1 containing viruses were consistent with the more severe pathology in biliary tracts, which transport digestive enzymes into the small intestine, suggesting that the injury seen at this anatomical site might be primarily responsible for the wasting and lethality phenotypes observed.

## Discussion

When newborn mice are infected i.p. or 4-day-old IFN deficient mice (either *Ifnar1*^-/-^*Ifngr1*^-/-^ or *Stat1*^-/-^ mice) are infected via the oral route, the RRV strain of simian RV has the unique ability to induce a severe systemic biliary obstructive disease characterized as acholic stool, oily fur, growth stunting, weight loss, and eventually death in 80–100% of mice [11,14,33]. A variety of other RV strains of human, murine, simian, bovine, and porcine origin do not induce this dramatic pathophysiology. RRV replication and severe pathology has been detected in the biliary tract and liver [11,14]. Of note, in IFN deficient mice, RRV replication and pathology can also be detected in the pancreas, however, viral replication in the pancreas had not been previously studied in the suckling mouse RRV infection model [11,33,34], which is a novel aspect of our current study that is discuss further below.

On the virus side, several RRV genes have been associated with RRV induced hepatic and systemic disease in suckling mice. Work from the Tiao lab linked RRV VP4 and, more specifically, the N-terminal domain of VP4, termed VP8* and an SRL-containing peptide within the VP5* domain, to the unique ability of the RRV strain to induce extra-intestinal infection and disease [18,19]. Using an intra-gallbladder injection model and selected reassortants derived from cell culture co-infection of RRV and the non-systemic disease causing bovine UK RV strain, we found that both RRV VP4 and NSP1 were necessary for RRV to replicate efficiently in biliary epithelial cells [20].

Due to recent advancements in RV reverse genetics [35], we now have the ability to construct a number of reassortant viruses by design, including reciprocal single RV NSP1 gene swaps between rRRV and rSA11, another simian RV strain, which had previously been showed to cause a significantly milder systemic disease syndrome [14,36]. This approach allowed us to more precisely re-interrogate the role of RRV NSP1 in extra intestinal replication and the systemic disease observed in newborn or suckling mouse pups with diminished IFN responsiveness. Here, we now show that IFN response deficient suckling mice orally administered rSA11 RV expressing RRV NSP1 develop a similar systemic disease as parental rRRV administered pups, characterized by a high proportion of infected pups developing signs and symptoms including acholic stool, oily fur, and weight loss (**Fig 1**). On the other hand, we observed that mice infected with rRRV with SA11 NSP1 developed signs and symptoms similar to the parental rSA11 virus, in which only acholic stool was observed without accompanying oily fur and weight loss. We did not use mortality as an experimental end point in most of these studies to reduce excessive suffering of the animals since the distinctive disease phenotypes of RRV and SA11 could be confirmed and quantified through alternative measurement of viral replication in extra-intestinal organs, tissue pathology, blood chemistry, and the disease phenotype of oily fur.

In addition to the differential severity of clinical symptoms described above, we found that mice infected either with RRV or SA11 expressing an RRV NSP1 had higher levels of viral replication in the liver and accompanying extrahepatic biliary tree and pancreas compared to SA11 or RRV with SA11 NSP1 (**Fig 3B and 3C**). However, that all RVs studies here were heterologous in the context of the murine RV infection model, the rate and duration of diarrhea disease, as well as the levels of fecal RV shedding among these groups were not correlated with the origin of NSP1s (**Figs 1 and 3A**). Of note, the two simian origin RRV and SA11 strains had previously been shown to induce comparable levels of diarrhea in suckling mice [14,37], the mechanism of which is likely multigenic. Hence, these studies support the conclusion that the parental origin of NSP1 primarily determines the differential ability of the two simian viruses to replicate in extra-intestinal organs and cause severe systemic disease.

Additionally, we generated a mono-reassortant of RRV with NSP1 derived from the bovine UK RV strain. Replacement of RRV NSP1 with UK NSP1 eliminated RRV’s ability to induce systemic disease, significantly reduced fecal shedding, and significantly diminished viral replication in both liver and pancreas. UK has previously been shown to replicate minimally in both mouse intestines and biliary epithelial cells [20]. Hence, the UK NSP1 (and probably NSP1s from other less enteric virulent heterologous RVs) severely restrict the ability of RRV to replicate in both the intestine and extra-intestinal organs and the ability to cause RV associated systemic disease. Hence the data presented here further supports the conclusion that RRV NSP1 is both necessary and sufficient for the induction of enhanced extra intestinal replication and systemic disease when expressed within the genetic background of either RRV and SA11 simian RV in suckling IFN deficient mice.

In this report, we also demonstrate that VP4s from both RRV and SA11 have very similar effects in determining the differential extra-intestinal infection and disease phenotype since a mono-reassortant rSA11 expressing RRV NSP1, which has a VP4 from rSA11, replicated to similar levels in the liver and pancreas as homologous rRRV (**Fig 3**). On the other hand, rSA11 with RRV VP4 did not show a significant enhancement of viral replication in the liver or pancreas nor did it cause the RRV associated systemic disease in mice (**Figs 1 and 3**). These findings complement a recent publication showing that the genetic origin of simian or bovine VP4 does not contribute significantly to the rotavirus associated host range restriction of intra-intestinal replication in the suckling mouse model [23]. However, VP4s from other RV strains have been shown to significantly modulate RV replication in biliary epithelial cells, extra intestinal organs and induction of systemic diseases [19,20]. Therefore, the role of VP4 in the development of biliary atresia is likely context dependent on the origin of the RV strains examined and its involvement may be affected by other viral gene products. In addition differences in the mouse genetic background may be important (Balb/c *vs*. 129sv).

The two simian origin RVs examined here, RRV and SA11, clearly caused different pathological changes in the extra-intestinal organs examined and were associated with variations in several systemic findings. Severe edema and inflammatory cell infiltrations were observed in intra- and extra-hepatic biliary tracts in rRRV, rSA11 and RRV or SA11 NSP1 monoreassortants (**Fig 6**). These inflammatory responses resulted in narrowing of the extra hepatic common bile ducts, and likely led to ductal blockage, acholic stools, and eventually higher serum bilirubin levels (**Fig 8A**).

RV induced inflammatory responses were found surrounding intra-hepatic portal triads (**Fig 5**), as previously reported [11,14]. However, RV infected cells were not limited to biliary ductal epithelium since RV antigens were also observed in non-epithelial cells in portal triads as well as areas in liver parenchyma (**S2A Fig**), suggesting immune cells and/or hepatocytes might also be infected. However, serum ALT levels were not significantly different among these groups and in the non-infected control group unlike previous reports [38]. It has been reported that bilirubin levels tend to elevate around day 7 post RRV infection, while ALT elevation occurs later after day 14 post infection [38]. This later elevation time might explain the similar levels of ALT observed in all the groups, including the non-infected control, in this study (**Fig 8D**). It should be noted that mild increases in blood ALT and aspartate aminotransferase levels are also not uncommon in RV-infected infants [39].

RV involvement in the pancreas had previously been reported only in the context of type 1 diabetes studies in nonobese diabetic mice [40]. However, RRV infection of the pancreas has not been examined in studies using the IFN-deficient suckling mouse model [34]. In addition to detecting viral replication (**Fig 3C**), we also detected pancreatic tissue damage in the rRRV and rSA11 with RRV NSP1 infected pups (**Fig 7**). Similar to RV infection in the liver, RV positive immunostained cells were detected in both pancreatic ductal epithelial cells and cells surrounding pancreatic ducts (**S2C Fig**). Viral induced pancreatic damage was not observed following rSA11 or rRRV with SA11 NSP1 infection, which is consistent with the absence of viral replication in the pancreas of mice infected with these viruses. Viral induced pancreatic damage was also indicated using functional pancreatic enzyme serum chemical assays. Elevations of serum amylase and lipase were shown in rRRV or rSA11 with RRV NSP1 infected mice (**Fig 8B and 8C**). Based on the levels of viral replication, pathological changes, and the results of serum chemistry tests, we hypothesize that the severe clinical manifestations of acholic stool, oily fur, weight loss and mortality seen in this disease model were caused by the combination of viral induced biliary tract, liver, and pancreatic injury.

RV NSP1 is a known virally encoded IFN antagonist that degrades host IRF3/5/7 and β-TrCP [25], resulting in a blunted anti-viral IFN response. Specific regulation of this response plays an important role in the highly species-specific character of RV infection [41]. We have shown that homologous wild-type murine RV replicates to approximately 10,000-fold higher titers in the mouse intestine than the heterologous RRV, and that this species specific phenotype is determined specifically by the presence of the murine RV NSP1 protein [15,42]. Using classically derived reassortant viruses, we also demonstrated that RRV is much less efficient in blocking host IFN response in the mouse intestine than murine RV and that the RRV derived NSP1 diminished the ability of homologous murine RV to efficiently replicate in mouse intestines [15]. More recently, we showed, using RV a reverse genetics approach, that murine-derived NSP1 is essential for efficient RV intestinal replication and diarrhea [22]. Paradoxically, although RRV is much less efficient than murine RV in blocking the host IFN response in the mouse intestine, the latter did not induce systemic disease mice, either in newborns or in IFN deficient suckling mice [14]. Since we performed these studies in *Stat1*^-/-^ mice, it is not certain what additional novel functions that RRV NSP1 possesses during RV replication in the absence of functional host IFN signaling *in vivo*. Even though RV NSP1 is not essential for viral replication *in vitro* [43], it may play an important role in modulating the efficiency of tissue specific viral replication and the severity of the innate immune response, especially inflammatory responses (*e*.*g*., TNF-α or IL-1 signaling) even in the absence of IFN activity [22]. In an initial attempt to address this question, we found that the biliary disease phenotype caused by RRV NSP1 was disrupted by deleting most parts of NSP1 (NSP1 deletion virus) but not disrupted by deleting the C-terminal IRF3 binding domain (ΔC16) (**Fig 2B–2D**). These results clearly show an uncharacterized function of NSP1 that warrants further study. The mechanisms responsible for viral strain specific NSP1 regulation of systemic RV replication, pathogenesis, and disease phenotypes remain to be elucidated in future studies.

From the standpoint of the host, IFN signaling deficiency appears to be a prerequisite for orally administered RRV to induce systemic disease in the suckling mouse model [14]. The role of IFN in the newborn RRV i.p. infection induced systemic disease model is presently not entirely clear. However, since this model system is very sensitive to the mouse age, the development related immaturity of the host IFN system in newborn mice may well play a key role. The levels of specific inflammatory cytokines and chemokines in the absence of IFN in the RRV oral infection model were not examined in detail in this study and are an important new area for future studies. Inborn errors of immunity such as STAT1 and STAT2 in human infants will also be of interest to examine in the context of biliary atresia development.

## Supporting information

S1 Fig Percentage of diarrhea, acholic stool, and biliary obstructive disease (acholic stool, oily fur, and growth retardation) in *Stat1*^-/-^ mice suckling mice orally infected with recombinant RVs encoding different domains of RRV NSP1. (A) Western blot images of IRF3 degradation in MA104 cells (multiplicity of infection of 3, 8 hours post infection). (B) rSA11 with RRV NSP1 R86* (RING-finger domain); (C) rSA11 with RRV NSP1 C179* (RING-finger domain and cytoskeleton binding domain); (D) rSA11 with RRV NSP1 N328* (RING-finger domain, cytoskeleton binding domain, and undefined domain); (E) daily weight changes; (F) Kaplan–Meier survival curves.
(TIF)

S2 Fig Immunofluorescent staining of liver and pancreas in *Stat1*^-/-^ suckling mice orally infected with rRRV or rSA11 at day 10 post infection. Tissues were stained with Alexa Fluor 488 labeled rabbit monoclonal antibody against CK7 (green), Texas Red labeled rabbit polyclonal antibody against RV (red), and DAPI (blue). (A) liver infected with rRRV; (B) liver infected with rSA11; (C) pancreas infected with rRRV; (D) pancreas infected with rSA11. Scale bar: 100 μm.
(TIF)

S3 Fig Profile of immune cells in the liver in *Stat1*^-/-^ suckling mice orally infected with mock, rSA11, and rSA11 expressing RRV NSP1. Flow cytometry of mononuclear cells harvested from day 10 post infection. (A) CD4 T cells; (B) CD8 T cells; (C) NK cells; (D) B cells. N = 3 in each group. * (P<0.05), ** (P<0.01), *** (P<0.01).
(TIF)
