# Supplementary figures and images for: Rhesus rotavirus NSP1 mediates extra-intestinal infection and is a contributing factor for biliary obstruction
Source: PLoS Pathog. 2024 Sep 30;20(9):e1012609. doi: 10.1371/journal.ppat.1012609 (PMC11476687; doi:10.1371/journal.ppat.1012609)

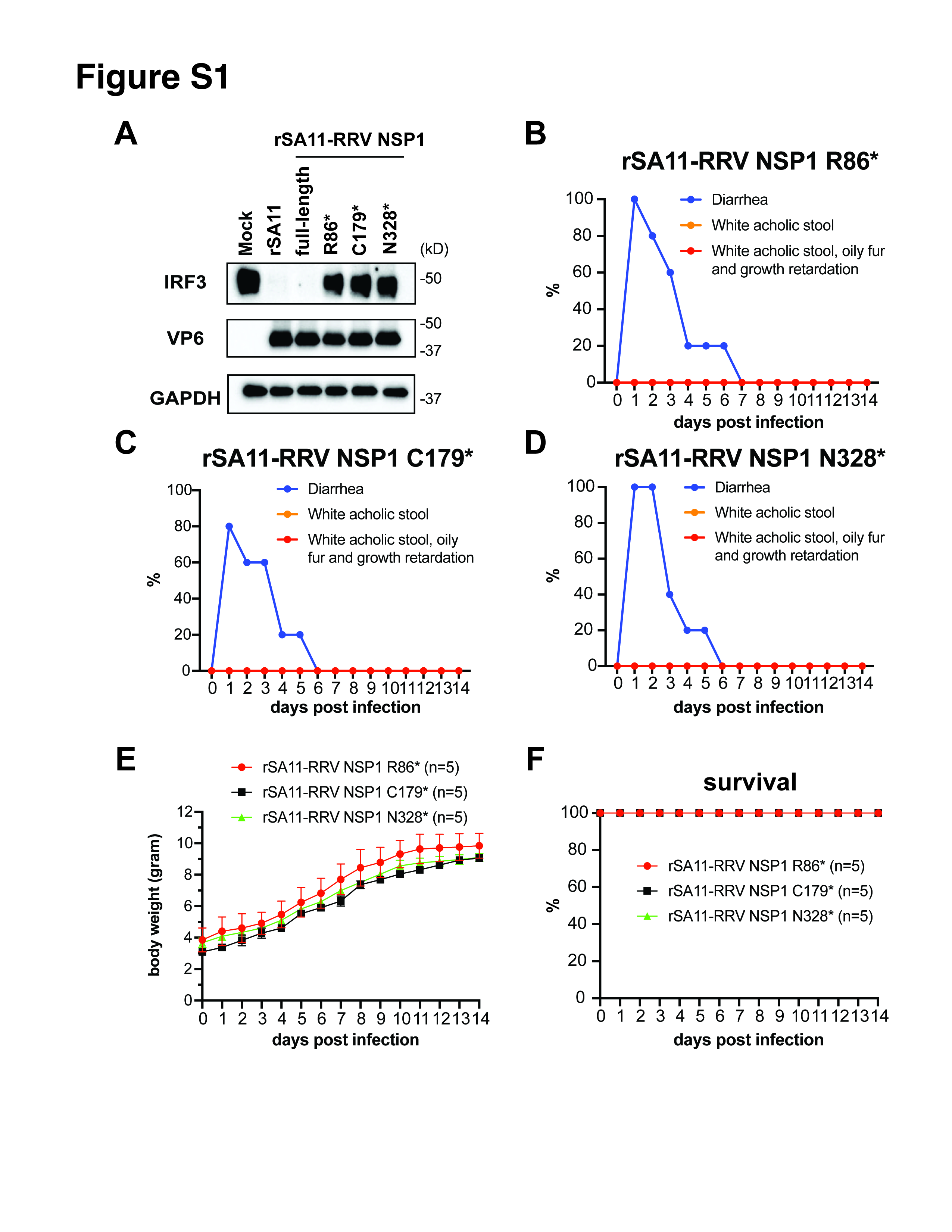

Supplement: S1 Fig — (A) Western blot images of IRF3 degradation in MA104 cells (multiplicity of infection of 3, 8 hours post infection). (B) rSA11 with RRV NSP1 R86* (RING-finger domain); (C) rSA11 with RRV NSP1 C179* (RING-finger domain and cytoskeleton binding domain); (D) rSA11 with RRV NSP1 N328* (RING-finger domain, cytoskeleton binding domain, and undefined domain); (E) daily weight changes; (F) Kaplan–Meier survival curves. (TIF) [file ppat.1012609.s001.tif]

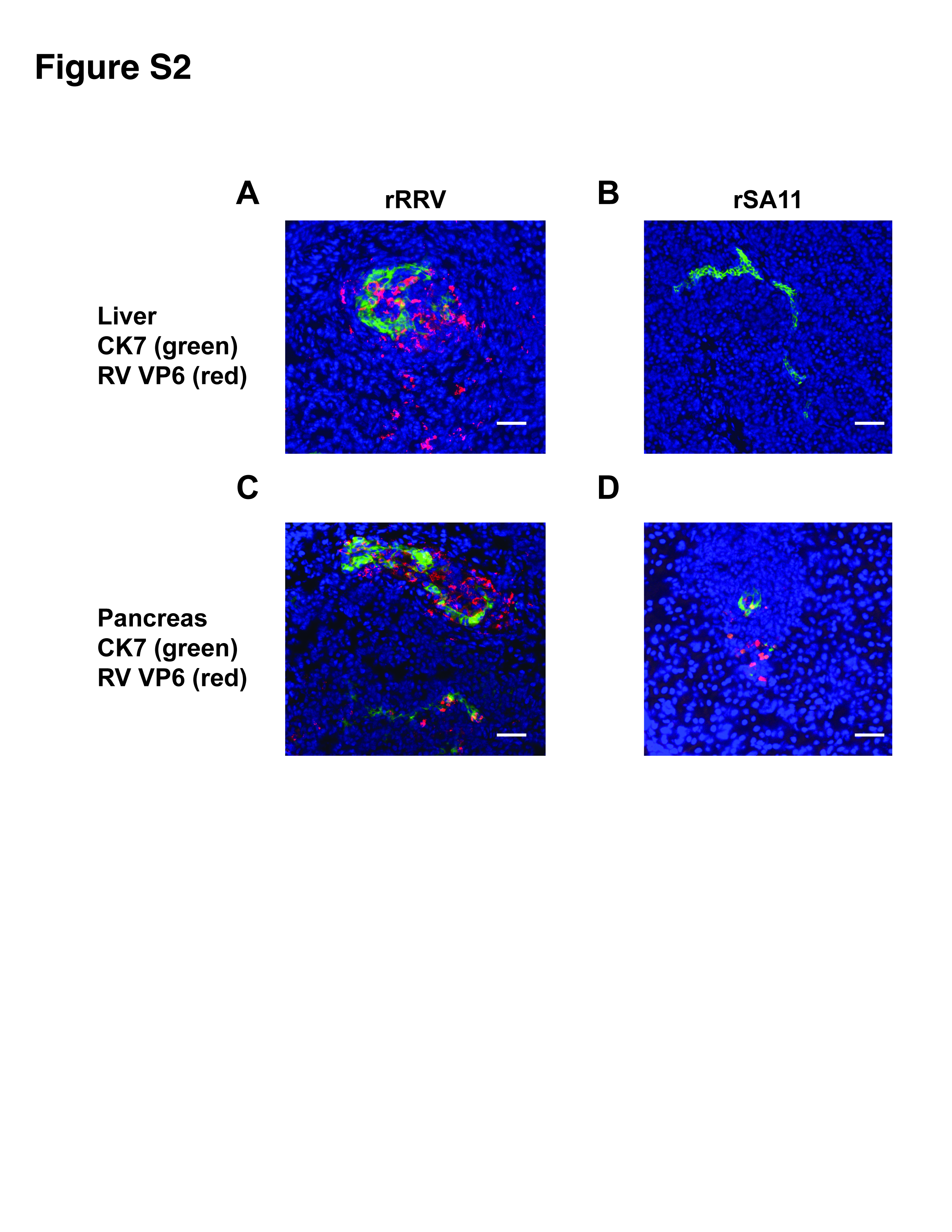

Supplement: S2 Fig — Tissues were stained with Alexa Fluor 488 labeled rabbit monoclonal antibody against CK7 (green), Texas Red labeled rabbit polyclonal antibody against RV (red), and DAPI (blue). (A) liver infected with rRRV; (B) liver infected with rSA11; (C) pancreas infected with rRRV; (D) pancreas infected with rSA11. Scale bar: 100 μm. (TIF) [file ppat.1012609.s002.tif]

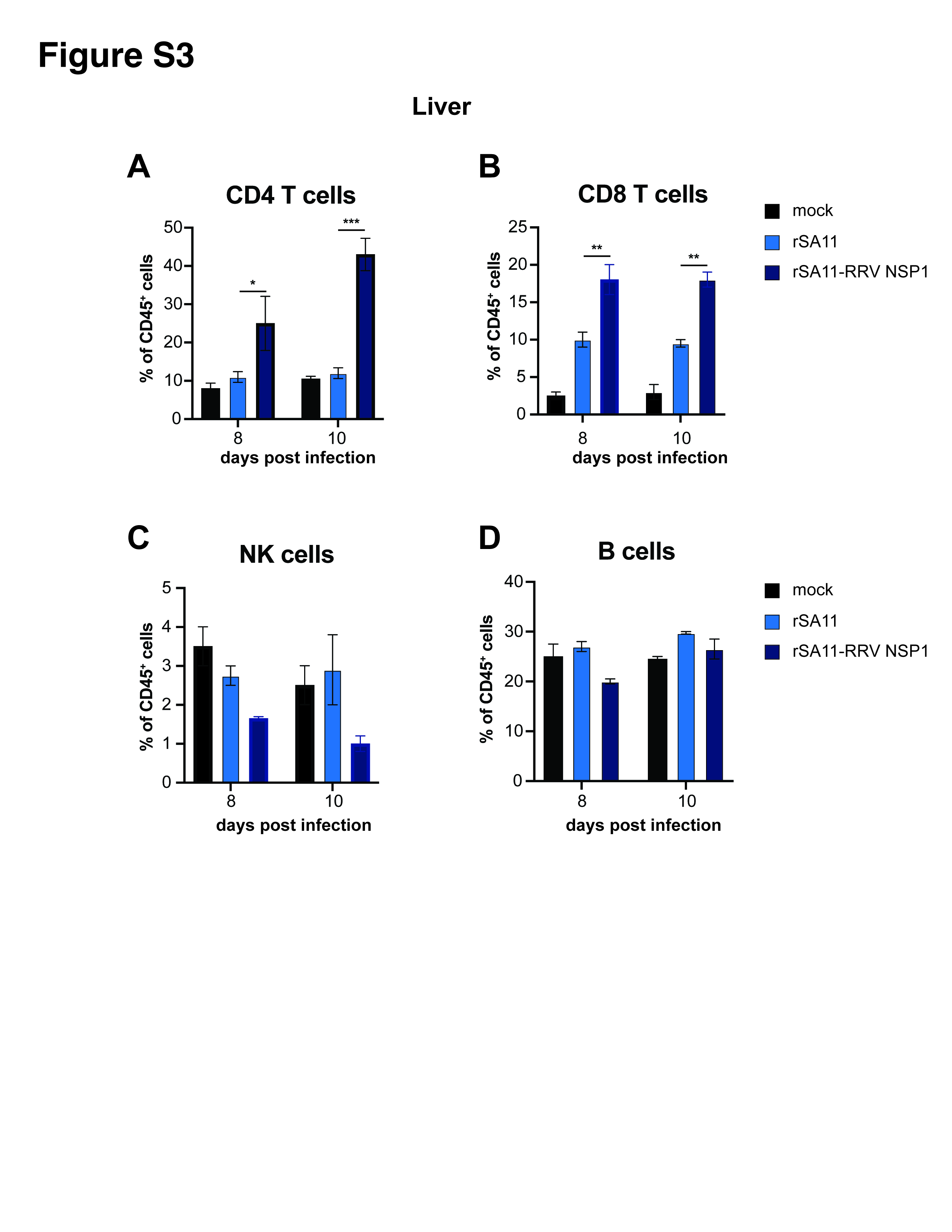

Supplement: S3 Fig — Flow cytometry of mononuclear cells harvested from day 10 post infection. (A) CD4 T cells; (B) CD8 T cells; (C) NK cells; (D) B cells. N = 3 in each group. * (P<0.05), ** (P<0.01), *** (P<0.01). (TIF) [file ppat.1012609.s003.tif]
